# Two subgroups of antipsychotic-naive, first-episode schizophrenia patients identified with a Gaussian mixture model on cognition and electrophysiology

**DOI:** 10.1038/tp.2017.59

**Published:** 2017-04-11

**Authors:** N Bak, B H Ebdrup, B Oranje, B Fagerlund, M H Jensen, S W Düring, M Ø Nielsen, B Y Glenthøj, L K Hansen

**Affiliations:** 1 Centre for Neuropsychiatric Schizophrenia Research, Mental Health Services Glostrup, University of Copenhagen, Copenhagen, Denmark; 2 Centre for Clinical Intervention and Neuropsychiatric Schizophrenia Research, Mental Health Glostrup, University of Copenhagen, Copenhagen, Denmark; 3 Department of Psychiatry, Brain Center Rudolf Magnus, University Medical Center Utrecht, Utrecht, The Netherlands; 4 Faculty of Health and Medical Sciences, Department of Clinical Medicine, University of Copenhagen, Copenhagen, Denmark; 5 Cognitive Systems, DTU Compute, Department of Applied Mathematics and Computer Science, Technical University of Denmark, Kongens Lyngby, Denmark

## Abstract

Deficits in information processing and cognition are among the most robust findings in schizophrenia patients. Previous efforts to translate group-level deficits into clinically relevant and individualized information have, however, been non-successful, which is possibly explained by biologically different disease subgroups. We applied machine learning algorithms on measures of electrophysiology and cognition to identify potential subgroups of schizophrenia. Next, we explored subgroup differences regarding treatment response. Sixty-six antipsychotic-naive first-episode schizophrenia patients and sixty-five healthy controls underwent extensive electrophysiological and neurocognitive test batteries. Patients were assessed on the Positive and Negative Syndrome Scale (PANSS) before and after 6 weeks of monotherapy with the relatively selective D_2_ receptor antagonist, amisulpride (280.3±159 mg per day). A reduced principal component space based on 19 electrophysiological variables and 26 cognitive variables was used as input for a Gaussian mixture model to identify subgroups of patients. With support vector machines, we explored the relation between PANSS subscores and the identified subgroups. We identified two statistically distinct subgroups of patients. We found no significant baseline psychopathological differences between these subgroups, but the effect of treatment in the groups was predicted with an accuracy of 74.3% (*P*=0.003). In conclusion, electrophysiology and cognition data may be used to classify subgroups of schizophrenia patients. The two distinct subgroups, which we identified, were psychopathologically inseparable before treatment, yet their response to dopaminergic blockade was predicted with significant accuracy. This proof of principle encourages further endeavors to apply data-driven, multivariate and multimodal models to facilitate progress from symptom-based psychiatry toward individualized treatment regimens.

## Introduction

Despite great progress in the characterization of specific functional and structural deficits associated with schizophrenia,^1^ the clinical diagnosis according to Diagnostic and Statistical Manual of Mental Disorder and International Classification of Diseases classifications still relies entirely on symptomatology rather than on objective, biological measures. A wide range of group differences ranging from genetic variation over brain structural and functional changes to deficits in early information processing and cognition have been reported in schizophrenia patients when compared with groups of matched healthy controls.^2, 3^

Although many of these neurobiological findings have been reproduced at a meta-analytical level,^4^ the great heterogeneity between studies suggests that schizophrenia is composed of several biologically different subgroups.^5^ The presence of subgroups implies that any given clinical sample of schizophrenia patients comprise multiple subgroups with different patterns of biological disturbances. Such ‘mixed' patient samples may hinder identification of effective, individually targeted clinical management. Moreover, the presence of subgroups impairs the development of novel treatment strategies, as potentially important clinical effects may be masked by unknown variance in the clinical sample of schizophrenia patients.^6, 7^ Finally, observations of deficits in schizophrenia patients are often confounded by effects of chronicity, substance abuse and previous treatment exposure. Subgroups of schizophrenia patients based on biologically valid, objectively measured markers have not yet been identified.

Deficits in early information processing and cognition are among the most robust findings in schizophrenia.^8, 9^ In early information processing, sensory input is filtered so that only relevant input reaches the conscious, cortical level. Early information processing can be estimated with electrophysiology using several paradigms, such as P50 suppression,^10^ the pre-pulse inhibition (PPI) of the startle response^11^ and mismatch negativity (MMN).^12^ Despite the presence of psychotic symptoms, cognition can be reliably assessed by neurocognitive tests, and pronounced cognitive deficits constitute core deficits in schizophrenia patients.^9^

Handling of complex data sets comprising multiple variables from several modalities requires the application of novel algorithms. ‘Machine learning' enables identification of patterns in complex data, which cannot be modeled by means of more classical statistical methods. Identified patterns can potentially be used to predict future data or outcomes. In essence, the underlying hypothesis for applying machine learning is that a measurable structure in data exists. Machine learning techniques can be divided into supervised and unsupervised learning. In supervised learning, the ‘label', which can be a class or an outcome, has to be known for each observation, and the supervised algorithm identifies the underlying structure in the data, which is associated with this label. The identified data structure can then be applied to predict future, independent observations for which the label is unknown. Unsupervised learning, on the other hand, is used to identify new and unknown structures in data. Specifically, unsupervised learning is useful in order to subgroup data if the labels, that is, classes or outcomes are unknown. To ensure generalizable results, data-driven machine learning techniques require valid unbiased methods such as cross-validation.^13^

Previous machine learning studies in schizophrenia patients have reported on subgroup structures (referred to as ‘profiles' or ‘biotypes') based on electrophysiology (for example, Van Tricht *et al.*,^14^ Turetsky *et al.*,^15^ Hall *et al.*,^16^ John *et al.*^17^), cognition (for example, Geisler *et al.*,^18^ Marder *et al.*^19^) or both,^20^ but first-episode, antipsychotic-naive patients have not been investigated.

The primary aim of the study was to identify potential subgroups of schizophrenia on measures of electrophysiology and cognition. For this, we applied an unsupervised machine learning algorithm in an unbiased cross-validation scheme for evaluation. The secondary aim was to investigate whether these subgroups were related to treatment response. For this, we applied a supervised machine learning algorithm including measures of psychopathology before and after treatment with a relatively selective dopamine D_2_ antagonist, amisulpride.

## Materials and methods

The study was conducted in accordance with the Declaration of Helsinki II, and approved by the Danish National Committee on Biomedical Research Ethics (H-D-2008-088). Clinical trials identifier: NCT01154829. All participants approved participation by signing informed consent.

### Participants

We included data from a multimodal first-episode study of antipsychotic-naive schizophrenia patients and healthy controls. The groups were matched on age, gender and socioeconomic status. Patients were excluded if they had a current diagnosis of drug dependency or were treated with antidepressants within the last month or during the study period. In addition, patients were asked to refrain from taking benzodiazepines the evening prior to a test day. At baseline, subjects underwent extensive assessments in multiple modalities, including cognitive and electrophysiological test batteries. Part of the data overlap with previous uni-modal publications on electrophysiology^21, 22, 23^ as well as publications on functional and structural magnetic resonance imaging,^24, 25, 26, 27^ oxidative stress^28^ and single-photon emission computed tomography data.^29^

After baseline assessments, the patients underwent 6 weeks of antipsychotic monotherapy with the relatively selective D_2_ receptor antagonist, amisulpride. Symptom severity in patients was measured with the Positive and Negative Syndrome Scale (PANSS).^30^ The subgrouping analyses in the current study included data on electrophysiology (baseline), neurocognition (baseline), PANSS (baseline and follow-up) and amisulpride dose (follow-up).

### Participants for analyses and imputation procedure

We included 69 antipsychotic-naive schizophrenia patients and 67 healthy controls. Three patients and two controls did not undergo the electrophysiological and cognitive test batteries. These five subjects were excluded. In addition, 39 subjects had one or more missing variables in electrophysiology or cognition data leaving 92 complete cases (41 patients and 51 controls). On the subset of subjects, who had one or more missing variables, we performed an imputation procedure.^31^ After imputation, the data set comprised 97 subjects (44 patients and 53 controls). The subgrouping analyses were performed on these 44 patients. Subsequent supervised machine learning analyses were based on the patients, who had PANSS baseline (*N*=43), follow-up (*N*=36) or both (*N*=35) (due to missing data for one PANSS baseline assessment).

A short description of acquisition and processing of cognitive and electrophysiological data is provided below. Details of the procedures are presented in Supplementary Material.

### Cognition

All participants were examined with a comprehensive neurocognitive test battery. The neurocognitive battery took ~2 h to complete and participants were allowed short breaks between tests. All tests were administered by research staff trained in standardized administration and scoring of the battery. Outcome variables from the following neurocognitive tasks were included from: Danish Adult Reading Test,^32^ Wechsler Adult Intelligence Scale III,^33^ Brief Assessment of Cognition in Schizophrenia^34^ and Cambridge Neuropsychological Test Automated Battery.^35^ Please, also see Supplementary Material.

### Electrophysiology

Participants were examined with the Copenhagen Psychophysiology Test Battery.^21, 22, 36^ The Copenhagen Psychophysiology Test Battery includes PPI, P50 suppression, MMN and selective attention paradigms in a fixed order. Tobacco use was not allowed 1 h before testing in order to avoid acute and/or withdrawal effects of nicotine.^37^ Participants were instructed to refrain from intake of caffeinated beverages at the day prior to testing. Testing was performed in a separate room with a sound level <40 dB situated adjacent to the control room. Participants were seated in a comfortable armchair and were instructed to keep movements to a minimum, keep their eyes fixed on a spot on the wall directly in front of them and stay awake. Auditory stimuli were presented by a computer running Presentation (Neurobehavioral Systems, Albany, NY, USA) software (soundcard: Creative Sound Blaster 5.1, 2008 Creative Technology, Singapore). Stimuli were presented binaurally through stereo insert earphones (Eartone ABR, 1996–2008 Interacoustics A/S, Assens, Denmark; and C and H Distributors, Milwaukee, WI, USA). The soft- and hardware audio settings were calibrated with an artificial ear (Brüel and Kjær, type 2133, Odin Metrology, Thousand Oaks, CA, USA).

Electroencephalography as well as electromyography recordings were performed using BioSemi hardware (Amsterdam, The Netherlands) using a cap with 64 active electrodes. For PPI, the eye-blink component was measured by recording electromyography activity from the right musculus orbicularis oculi with two electrodes. The first of these was aligned with the pupil, the other positioned just laterally. BESA software (version 5.2.4, MEGIS Software, Gräfelfing, Germany) was used for further processing of the data. A background noise consisting of 70 dB white noise was used in all paradigms. Please, also see Supplementary Material.

### Statistical analyses

Analyses were performed in MATLAB (The MathWorks, Natick, MA, USA) using the Statistics and Bioinformatics Toolbox Release 2013a.

#### Identification of subgroups

**Figure 1:**
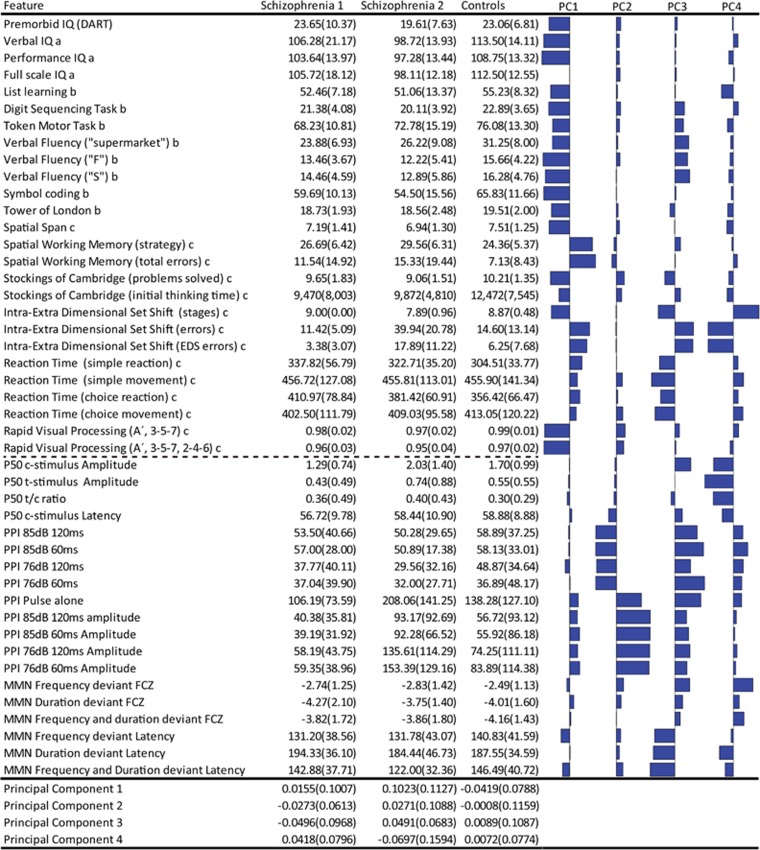
Cognitive and early information processing data. Mean (s.d.) for the two subgroups and controls. The weights for each feature on the four principal components (PC1-4) are shown as bar charts. Cognitive features from Danish Adult Reading Test (DART); ^a^Wechsler Adult Intelligence Scale III; ^b^Brief Assessment of Cognition in Schizophrenia; ^c^Cambridge Neuropsychological Test Automated Battery. Electrophysiological features from P50 suppression (P50); pre-pulse inhibition (PPI) of the startle effect; mismatch negativity (MMN).

In order to identify potential subgroups of schizophrenia, we applied unsupervised machine learning analyses on 26 cognitive and 19 electrophysiological variables, denoted ‘features' (Figure 1).

A probabilistic principal component analysis based on the 45 standardized (scaled to a mean of zero and unit variance) features from all subjects with complete data sets (*N*_complete_=92) was performed, identifying the 45-dimensional (D) principal component space. The whole sample was used in this step in order to be able to compare the healthy subjects with patients in a space unbiased toward patients or controls and avoid variance inflation.^38^ The 45 components were sorted so that the first component explained most variance in the data. Each of the subsequent components explained most of the remaining variance.

**Figure 2:**
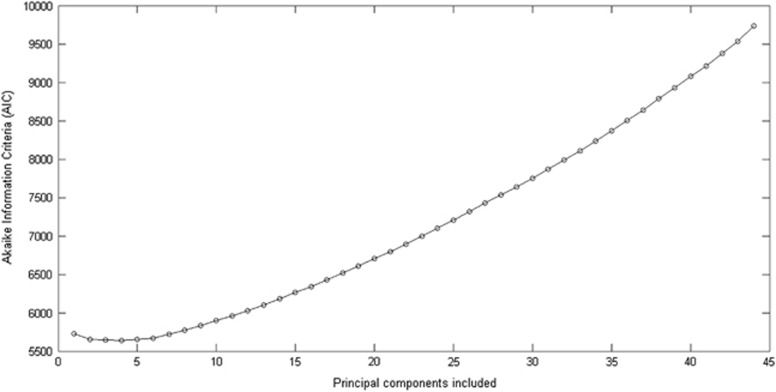
AIC indicating likelihood for number of principal component dimensions. The minimal AIC value is attained at D=4, models based on larger or smaller dimensions provide poorer fits to the test data. AIC, Akaike information criteria.

To identify the statistically distinct PCA subspace of the 45D principal component space, we applied the Akaike information criterion (AIC).^39^ AIC is an analytic and asymptotically unbiased estimator of the cross-validation deviance (mean log-likelihood of test data). The asymptotic estimator is appropriate, as we apply this step for the combined cohort of patients and controls.^40^ AIC was calculated for dimensions D=2, 3, 4, …, 45 to determine the number of principal components, which best described the data. AIC identified an optimal number of four components, which were then used in the further analyses (Figure 2).

On the subset of subjects (*n*=39), who had one or more missing variables, we performed an imputation procedure.^31^ This method provides an estimate of the imputation error and variance, by simulating missing values in subjects with complete data sets and weighing these by similarity to subjects with missing values. Subjects with missing values and an estimated error below 0.05 (s.d.=0.1 in all four PCs) in the 4D principal component space were imputed and included in the analyses, while subjects with higher estimated imputation errors were excluded. After the imputation procedure, the analyzed data set comprised 97 subjects (44 patients and 53 controls).

A Gaussian mixture model (GMM) was used to identify group structure (patients only) in the 4D principal component space, using leave-one-out cross-validation^41^ based on subjects to estimate the optimal number of groups. The GMM is an unsupervised clustering algorithm that fits data as a number of ‘structures' in the Gaussian mixtures. Each structure in the Gaussian mixture represents a subgroup in the data. The structures should not be confused with the principal components that determine the dimensions in which the subgroups are found. The GMM was run on patients alone (*N*=44), as we aimed to identify subgroups within patients rather than classify patients from controls. To provide an unbiased estimate of test error, the mean negative log likelihood was estimated for a range of groups, *K*=1,…, 10 in each cross-validation fold.

To further characterize the identified subgroups, we applied *χ*^2^-test for gender and use of alcohol, tobacco, cannabis or benzodiazepines. Patients fulfilling criteria ‘never tried' or ‘tried a few times' regarding each item were defined as ‘non-users'. Patients fulfilling criteria ‘regular use' or ‘abuse' were defined as ‘users'. We applied independent *t*-tests to test for differences in age, PANSS subscores and amisulpride dose to explore psychopathological subgroup differences. Specifically, we included amisulpride dose at follow-up, positive, negative, and general subscores from baseline, follow-up and change scores (baseline−follow-up; Table 1).

#### Prediction of treatment response

In order to investigate whether the two identified subgroups were related to treatment response, we used PANSS subscores (positive, negative, general) from baseline, follow-up (6 weeks) and changes in PANSS subscores (baseline−follow-up), which represented the treatment effect. For this, we performed three separate linear support vector machines (SVMs)^42^ based on the PANSS subscores (baseline, follow-up or changes; Table 1) with the ‘*C*' parameter set to 100. The SVM algorithm classifies the data regarding label by finding the multidimensional hyperplane with the largest margin that separates the labels in the input space. The measure of ‘accuracy' estimates whether the PANSS scores can predict the identified subgroups. This can be considered an external validation of the subgroups and thereby indicate whether the subgroups have potential clinical relevance. Leave-one-out cross-validation was used to obtain an unbiased estimator of the predictability and the strength of the evidence was evaluated by permutation test. One thousand permutations of group labels were performed, refitting the SVM to randomized labels and testing accuracy in each.

### Code availability

Computer code available upon request.

## Results

Patients (*N*=44) and controls (*N*=53) were well matched on age (*t*=0.222, *P*=0.825) and gender (*χ*^2^<0.001, *P*=0.983). At follow-up, the mean amisulpride dose was 280.3 (159.6) mg per day.

### Identification of subgroups

The first principal component identified with probabilistic principal component analysis loaded on all cognitive features thus represents the overall cognitive capacity (also referred to as ‘Spearman's G').^43^ The second principal component loaded primarily on electrophysiological features, specifically the features from PPI of the startle reflex. The third and fourth principal components involved features from both modalities. More specifically, the third component principal component loaded on executive functions and reaction time from the neurocognitive modality and percent inhibition in PPI and latency in MMN. The fourth component loaded on intra-extra dimensional set shifting and all variables from P50 suppression. See Figure 1 for weights of the four principal components.

**Figure 3:**
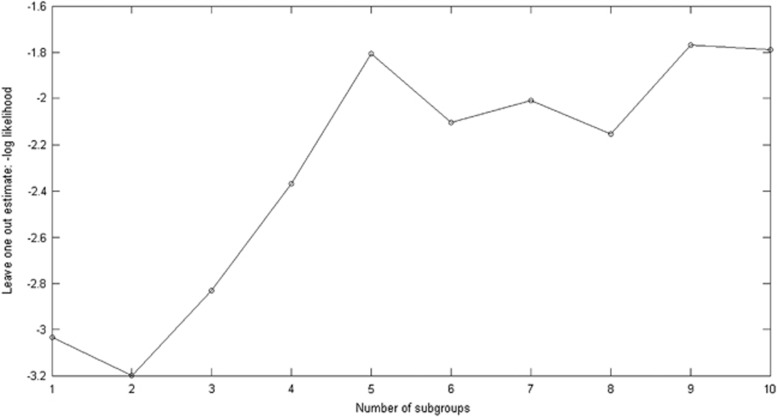
Leave-one-out estimate of test error (negative log likelihood). Calculated for model dimension, that is, number of subgroups=1,2, …, 10. Lowest value at model dimension=2, indicating that a model with two subgroups best-fit the test data. Values on *Y* axis are arbitrary.

As indicated by the lowest negative log-likelihood score, the cross-validation of the GMM indicated that a separation into two subgroups of patients provided the most generalizable model, hence the model that best-fit the test data (Figure 3). ‘Subgroup 1' consisted of 26 patients and ‘Subgroup 2' consisted of 18 patients. Data on the two patient subgroups and the controls are displayed in Figure 1.

Subgroup 1 had, compared to subgroup 2 lower values in PC 1 (representing higher cognitive capacity), lower values in PC 2 (indicating lower PPI and higher PPI amplitudes), and lower values in PC 3 (indicating increased reaction times and increased MMN latency). Conversely, subgroup 1 had higher values in PC 4 (indicating better performance in set-shifting and higher amplitudes in P50 suppression) compared to subgroup 2.

We found no significant differences in between the two patient subgroups with regard to age, gender, PANSS subscores, amisulpride dose, or use of alcohol, tobacco, cannabis or benzodiazepines (Table 1).

### Prediction of treatment response

**Figure 4:**
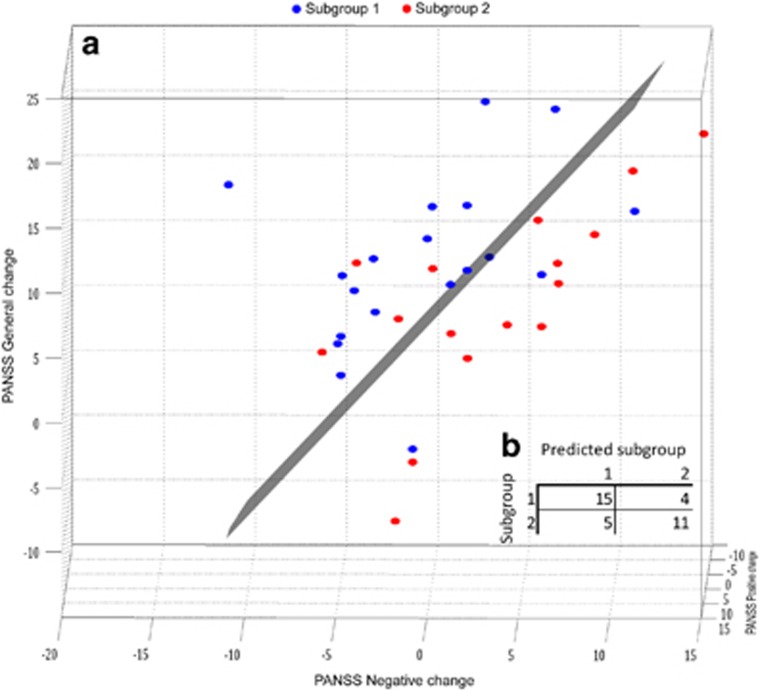
(**a)** The two subgroups in 3D PANSS change subscores with SVM decision surface. The decision surface is determined by PANSS negative and PANSS general subscore changes while PANSS positive change scores seems to have limited to no influence. (**b**) Confusion matrix presenting actual vs predicted group. PANSS, Positive and Negative Syndrome Scale; SVM, support vector machines.

SVM analyses of the predictive value of psychopathology showed that PANSS baseline subscores reached an accuracy of 67.4% (Table 1), which were significantly (*P*=0.017) higher than 58.1% in ‘baseline accuracy'. ‘Baseline accuracy' is defined as the accuracy for the simplest classification rule, that is, predicting the majority class for each observation. With PANSS follow-up subscores, SVM reached 50% compared to baseline accuracy of 55.6%. Using changes in PANSS subscores (baseline−follow-up), that is, the treatment effect, the SVM analysis reached an accuracy of 74.3%, significantly (*P*=0.003) higher than baseline accuracy (54.2% Figure 4).

## Discussion

Our multivariate analyses of multimodal non-biased data in a sample of first-episode antipsychotic-naive schizophrenia patients support the notion that the clinical diagnosis of schizophrenia encompasses biologically separable subgroups. Specifically, our data suggest the presence of two distinct subgroups of schizophrenia with regard to early information processing and higher cognitive functions. Univariate analyses showed no significant differences in demographic or clinical data at baseline or at follow-up. However, the SVM analyses showed that subgroup status can significantly be associated with the treatment effect after 6 weeks. Moreover, subgroups status was also associated with baseline PANSS subscores.

The change in PANSS scores reflects the overall clinical treatment response on positive, negative and general symptoms after 6 weeks of amisulpride monotherapy. Specifically, the accuracy of 74.3% was driven primarily by differences in PANSS negative and general scores (Table 1). Interestingly, subgroup 2 tended to improve in negative symptoms, whereas negative symptoms in subgroup 1 did not change after treatment. Amisulpride is the only antipsychotic compound that is approved for treatment of negative symptoms, yet the results from subsequent clinical studies have been inconsistent (for meta-analysis see Leucht^44^). A reduction in negative symptoms of three points on the PANSS scale represents the ‘clinical gain'. The modest clinical gain compared with the ‘high cost' of the present data acquisition and analyses do not support direct implementation of electrophysiological- and cognitive examinations as part of the routine work-up to predict treatment response to amisulpride. Nevertheless, the previously observed variable clinical trajectories in the response of amisulpride on negative symptoms^45^ may be explained by subgroups as identified in the current study.

To our knowledge, this is the first study applying both cognitive and electrophysiological measures in order to subgroup first-episode schizophrenia patients. One previous study identified three ‘biotypes' across schizophrenia, bipolar disorder and schizoaffective disorder using the same type of measures.^20^ Another study identified five subgroups of chronic schizophrenia patients based on aggregate cognitive scores of seven *a priori* defined domains.^46^ These studies investigated different diagnostic categories and included chronic, antipsychotic-treated patients and have in essence addressed the validity of the current diagnostic criteria.^47^ To our knowledge, the current study is also the first to demonstrate that more homogeneous samples of antipsychotic-naive, first-episode schizophrenia patients display both biologically and clinically relevant subgroup structures.

The two subgroups of schizophrenia identified in this study were identified among 44 first-episode schizophrenia patients. Although, effects of chronicity or previous antipsychotic exposure are ruled out, the findings call for replication in larger, independent samples. Unsupervised algorithms as used in this study usually require more observations than supervised algorithms to reach stable models.^48^ In machine learning terms, our number of observations is relatively low, and stresses the importance that the optimal number of latent variables and the optimal number of subgroups is identified properly, as recommended in a recent comprehensive review.^49^ The approach in the present study follows these guidelines. Another risk with unsupervised machine learning is that the structures/subgroups identified with might be associated with irrelevant traits, for example, head size, rather than the relevant traits, for example, clinical outcome. Our identified subgroups seem clinically relevant. Although supervised learning can generalize known statistical relations to new data, we have demonstrated the explorative potential of unsupervised learning to identify new generalizable statistical structure.

In the present study, we have only included data from two commonly used modalities in schizophrenia research (cognition and electrophysiology). Clearly, inclusion of more independent modalities may refine the structure and number of subgroups of schizophrenia patients. For example, functional magnetic resonance imaging has been shown to contain information to identify subgroups in schizophrenia.^50^ These modalities could include genetic variability, magnetic resonance imaging and *in vivo* receptor imaging with positron emission tomography or single-photon emission computed tomography. Moreover, ‘treatment response' in the context of this study is limited to 6 weeks, and the individual long-term course of illness may be subject to other trajectories. Finally, prediction of treatment response to other antipsychotic compounds than amisulpride, cannot be inferred from this study.

Overall, this proof of principle study supports the presence of biological, clinically relevant subgroups of schizophrenia and implies that stratification of patients is required to recognize specific treatment needs in individual subgroups. The current results encourage further endeavors to apply data-driven, multivariate and multimodal models to facilitate progress from symptom-based psychiatry toward individualized treatment regimens.

## Figures and Tables

**Table 1:** Demographic and clinical data on controls and the two identified subgroups of patients: PANSS data, baseline, follow-up and difference

*Measure*	*Group*	N	*Mean (s.d.)*	P	*SVM Acc*	*SVM p*
	Controls	53	30/23			
Gender (m/f)	1	26	17/9	0.168	—	
	2	18	8/10			
	Controls	53	24.79 (5.98)			
Age	1	26	23.46 (4.55)	0.197	—	
	2	18	26.06 (7.39)			
PANSS positive, B	1	25	19.92 (3.93)	0.727		
	2	18	20.39 (4.80)			
PANSS negative, B	1	25	20.32 (6.48)	0.545	0.674	**0.017**
	2	18	21.67 (7.99)			
PANSS general, B	1	25	41.64 (7.84)	0.380		
	2	18	39.22 (10.03)			
PANSS positive, Fu	1	20	13.10 (3.75)	0.091		
	2	16	15.31 (3.84)			
PANSS negative, Fu	1	20	18.15 (6.05)	0.916	0.500	
	2	16	17.94 (5.84)			
PANSS general, Fu	1	20	29.95 (8.13)	0.753		
	2	16	30.81 (8.10)			
PANSS positive, B-Fu	1	19	6.42 (4.90)	0.401		
	2	16	5.13 (3.95)			
PANSS negative, B-Fu	1	19	−0.37 (5.20)	0.056	0.743	**0.003**
	2	16	3.31 (5.79)			
PANSS general, B-Fu	1	19	10.47 (6.82)	0.187		
	2	16	7.13 (7.88)			
Amisulpride doses, Fu	1	17	276.47 (146.97)	0.890	—	
	2	16	284.38 (176.75)			
Alcohol (users)	1	26	21	0.506	—	
	2	18	13			
Tobacco (users)	1	26	17	0.307	—	
	2	18	9			
Cannabis (users)	1	26	9	0.189	—	
	2	18	3			
Benzodiazepines (users)	1	26	0	—	—	
	2	18	0			

Abbreviations: B, baseline; Fu, follow-up; PANSS, Positive and Negative Syndrome Scale; SVM, support vector machine.

*P*-value for the *t*-test for the difference between schizophrenia subgroups (*χ*^2^-test for gender and substance use). Support vector machine accuracy (SVM Acc) and *P*-value from the permutation test for significance of the SVM (SVM p). Significant *P*-values (*P*<0.05) are in bold.
