# Supplementary material for: Two subgroups of antipsychotic-naive, first-episode schizophrenia patients identified with a Gaussian mixture model on cognition and electrophysiology
Source: Transl Psychiatry. 2017 Apr 11;7(4):e1087–. doi: 10.1038/tp.2017.59 (PMC5416700; doi:10.1038/tp.2017.59)
Supplement: Supplementary Information [file tp201759x1.docx]

***Cognitive tasks***

The test battery comprised tests assessing premorbid and current intelligence, and specific neuropsychological functions using the Brief Assessment of Cognition in Schizophrenia (BACS) and the Cambridge Neuropsychological Test Automated Battery (CANTAB).

Premorbid intelligence was estimated using the Danish Adult Reading Test (the Danish version of the National Adult Reading test), which has been shown to be a valid estimate of premorbid intelligence in both healthy populations and schizophrenia patients (1). The DART consists of 50 irregular words that the subject is asked to pronounce. The outcome measure is the total number of correctly pronounced words.

Current intelligence was estimated using 4 subtests from the WAIS-III (Vocabulary, Similarities, Block design, and Matrix reasoning) which combined have shown a high correlation with a full-scale WAIS-III IQ (2). The vocabulary and similarities subtests assess verbal comprehension and conceptualization, while block design and matrix reasoning assess visuo-constructive comprehension and skills. Scores are standardized according to Danish normative data; the two verbal scores combined and multiplied to represent the verbal scale, and the two non-verbal scores are combined and multiplied to represent the non-verbal scale. The combined score from all four subtests comprise the estimated full-scale IQ.

The BACS was selected as a brief battery assessing verbal memory, processing speed, planning, motor skills, working memory. The BACS has a shown a high correlation with a much longer neuropsychological battery (3). List learning assesses verbal memory and learning. Across five trials, a list of fifteen words are read aloud to the subject, who is asked to recall as many words as possible immediately after. The outcome measure is the total number of words recalled across the five trials. In the Digit sequencing task, numbers are read aloud, and the subject is asked to repeat the numbers, ordered from the lowest to the highest. The number sequences are of increasing length across the test. Digit sequencing task assesses working memory, and the outcome measure is the total number to correct number sequences. The Token motor task assesses motor skills. One hundred tokens are placed on a table in front of the subject, who is asked to place them in a container as quickly as possible. The outcome measure is the number of corrected placed tokens within 60 seconds. Verbal fluency is assessed using the category and letter fluency tests. Subjects are given 60 seconds to state as many words as possible according to specific rules. The rules are words beginning with the letter ‘S’ or ‘F’, and items that can be found in a supermarket, and the outcome measure is the number of correct words according to each rule. Symbol coding assesses processing speed and motor skills. Subjects are given 90 seconds to fill in symbols below a row of numbers according to a key showing the corresponding number-symbol pairs. The outcome measure is the correct number of items. The Tower of London assesses planning, which is an aspect of executive functions. Pictures of coloured balls arranged on pegs are shown in a booklet. The subject is asked to compare two pictures and state how many moves would be required for the balls in one picture to be arranged identically to the balls in the other picture. The outcome measure is the number of correct responses.

In order to examine more specific neuropsychological functions, subtests previously shown to be sensitive to deficits in schizophrenia were selected from the CANTAB battery, e.g. (4). CANTAB is a computerized battery using a touch-sensitive screen. Spatial Span assesses the capacity of spatial working memory. Boxes are shown on the screen that change colour in a pre-specified order, after which the subject is required to touch the boxes in the same order. The level of difficulty (i.e. the number of boxes that change colour) increases throughout the test. The outcome measure is the number of boxes correctly remembered (i.e. the spatial span). In the Spatial working memory task, a number of boxes are shown on the screen. The subject is asked to find a blue token in each of the boxes, with only one blue token hidden at a time, which requires strategic working memory for efficient problem solving. The outcome measures are the strategy score (calculated across all trials) and the total number of errors made. The Stockings of Cambridge assesses efficiency of planning, which is a sub-component of executive functions. Coloured balls are shown, placed in two arrays in different positions. The subject is asked to move the balls in one of the array using as few movements as possible, in order for the display of the arrays to match. The outcome measures are the number of problems solved using the minimum number of moves, and the time taken to plan the movements (i.e. the time from the presentation of the array on the screen, until the subject first touches the screen). Intra-Extradimensional set shifting task assesses attentional set shifting, which is a sub-component of executive functions. Across nine stages of increasing difficulty, subjects are required to utilize feedback from the computer to change responses as rules continuously change. The outcome measures chosen are the number of stages completed, the total number of errors made (adjusted for possible stages that were not completed), and the number of errors made at the more difficult extra-dimensional shift stage. The Reaction time test assesses the reaction time and motor speed at which a subject responds to a target in a predictable location and in an unpredictable location. The outcome measures are reaction time (time taken to release a press pad) and motor time (time taken from releasing the press pad to touching the screen) in both a predictable location (simple reaction time and simple motor time) and unpredictable location (complex reaction time and complex motor time). Rapid visual information processing is a measure of sustained attention. Digits from 2-9 are displayed in a pseudo-random order on the screen, requiring subjects to respond on a press pad when a pre-designated sequence of numbers is displayed, one after the other. The sequences across the two versions of the test are 3-5-7; and 3-5-7, 2-4-6. The outcome measure is the signal detection measure of sensitivity to target, regardless of response tendency (A’), calculated based on probability of hits and the probability of false alarms.

***Electrophysiology paradigms***

Sensory gating was measured with a P50 suppression paradigm. Three experimental blocks, each with 40 pairs of stimuli (trials) with a fixed inter-trial interval of 10 s and an inter-stimulus interval (ISI) of 500 ms. Stimuli consisted of white noise bursts of 80 dB with a duration of 1.5 ms and instantaneous rise time. Processing of the P50 suppression data started with correction for eye movement by applying the surrogate model of BESA. The data were then epoched at -100 ms to 400 ms to each stimulus. Subsequently, non-paradigm related artifacts, e.g. due to movement were excluded by removing all epochs from the data where maximum and minimum amplitude differences exceeded 150 μV. The averaged epochs were then filtered between 1.6 and 70 Hz. P50 amplitudes for each subject were scored using the Cz electrode with average reference. The P50 waveform was defined as the largest trough-to-peak amplitude within 40–90 ms following the first (conditioning or ‘C’) stimulus in each paired trial. For the second (testing or ‘T’) stimulus the P50 amplitude was identified as the largest trough-to-peak amplitude within ±10 ms of the latency of P50 peak found for the C stimulus. P50 suppression was calculated as the ratio ‘T/C.’

Assessment of PPI and habituation started with 5 minutes of acclimation to background noise (70 dBa white noise), after which three experimental blocks of stimuli were superimposed on the background noise. The PPI assessments started with 5 minutes of acclimation to the background noise (70 dB white noise) after which three experimental blocks of stimuli were super-imposed on the background noise. The startle pulses were bursts of white noise with intensity: 115 dB and duration of 20ms, instant rise and fall time. The prepulses were bursts of white noise with intensities of 76 or 85 dB with a duration of 20 ms. Stimulus onset asynchrony (SOA) in prepulse-pulse trials was either 60 or 120 ms while intertrial intervals were randomized between10 and 20 s. This resulted in four types of trials based on the combination of SOA and intensity (60 ms/76 dB, 60ms/85 dB, 120 ms/76 dB, 120 ms/85 dB). Blocks 1 and 3 consisted of eight pulse-alone trials. Block 2 consisted of 50 trials to assess PPI, ten of each type of trial and ten pulse alone trials in a pseudo-randomized order (pulse alone trials were never in direct succession of each other). PPI data were filtered offline between 25 and 250 Hz. Then the startle amplitude for each type of trial was scored as the highest absolute amplitude between 20–100 ms after the pulse: PPI was defined as (1-($\frac{Prepulse-Pulse amplitude}{Pulse alone amplitude}$) *100%).

During the MMN paradigm subjects were asked to ignore all stimuli and to watch a muted video on a screen in front of them (a nature documentary). The paradigm consisted of 1800 binaurally presented stimuli with an ISI randomized between 300 and 500ms in a single run. All stimuli had an intensity of 75 dB. The duration of the paradigm was approximately 12 minutes. Four types of stimuli were used ̴$\sim$82% were standard tones with a frequency of 1000 Hz (50ms), $\sim$6% were deviant tones with frequency of 1200 Hz (50ms), $\sim$6% were deviant tones with a frequency of 1000 Hz (100ms), and $\sim$6% were deviant tones with a frequency of 1200 Hz (100ms). MMN was assessed at electrodes FCz, Fz and Cz, but only data from the electrode, where maximum amplitude was reached (electrode FCz) was used. First, data was resampled from the original 4kHz to 250 Hz to allow easier file handling. Second, the data were corrected for eye-artifacts by using the adaptive method of BESA. Third, the data were epoched (from 100ms prestimulus to 900ms poststimulus) and corrected for movement (or other paradigm unrelated) artifacts by removing all epochs from the data where maximum and minimum amplitude differences exceeded 75 µV in a time window between 0 and 500ms of these epochs. Subsequently, the data were filtered (low-pass set to 40 Hz, 24 dB/octave), after which MMN for each of the 3 deviant types was expressed as the average ERP of a subject to deviant stimuli, subtracted with the average ERP of this subject to standard stimuli. MMN amplitudes were then scored as the maximum negative voltage within a window between 50 and 275ms.

***Supplementary References***

1. O’Carroll R, Walker M, Dunan J, Murray C, Blackwood D, Ebmeier KP, et al. Selecting controls for schizophrenia research studies: the use of the national adult reading test (NART) is a measure of pre-morbid ability. Schizophr Res. 1992;8(2):137–41.

2. Axelrod BN. Validity of the Wechsler abbreviated scale of intelligence and other very short forms of estimating intellectual functioning. Assessment [Internet]. SAGE Publications; 2002 Mar;9(1):17–23. Available from: http://www.ncbi.nlm.nih.gov/pubmed/11911230

3. Keefe RS., Goldberg TE, Harvey PD, Gold JM, Poe MP, Coughenour L. The Brief Assessment of Cognition in Schizophrenia: reliability, sensitivity, and comparison with a standard neurocognitive battery. Schizophr Res. 2004;68(2):283–97.

4. Pantelis C, Brewer W. Neurocognitive and neurobehavioural patterns and the syndromes of schizophrenia: role of frontal-subcortical networks. In: Pantelis C, Nelson HE, Barnes TRE, editors. Schizophrenia a neuropsychological perspective. Chichester: John Wiley & Sons Ltd.; 1996. p. 317–43.
